# Insights into the evolution of symbiosis gene copy number and distribution from a chromosome-scale *Lotus japonicus* Gifu genome sequence

**DOI:** 10.1093/dnares/dsaa015

**Published:** 2020-07-13

**Authors:** Nadia Kamal, Terry Mun, Dugald Reid, Jie-Shun Lin, Turgut Yigit Akyol, Niels Sandal, Torben Asp, Hideki Hirakawa, Jens Stougaard, Klaus F X Mayer, Shusei Sato, Stig Uggerhøj Andersen

**Affiliations:** 1 Helmholtz Zentrum München, German Research Center for Environmental Health, Plant Genome and Systems Biology, 85764 Neuherberg, Germany; 2 Department of Molecular Biology and Genetics, Aarhus University, DK-8000 Aarhus C, Denmark; 3 Graduate School of Life Sciences, Tohoku University, Sendai 980-8577, Japan; 4 Kazusa DNA Research Institute, Kisarazu, Chiba 292-0816, Japan; 5 School of Life Sciences, Technical University Munich, Munich, Germany

**Keywords:** legume, symbiosis, rhizobium, comparative genomics, expression atlas

## Abstract

*Lotus japonicus* is a herbaceous perennial legume that has been used extensively as a genetically tractable model system for deciphering the molecular genetics of symbiotic nitrogen fixation. Our aim is to improve the *L. japonicus* reference genome sequence, which has so far been based on Sanger and Illumina sequencing reads from the *L. japonicus* accession MG-20 and contained a large fraction of unanchored contigs. Here, we use long PacBio reads from *L. japonicus* Gifu combined with Hi-C data and new high-density genetic maps to generate a high-quality chromosome-scale reference genome assembly for *L. japonicus*. The assembly comprises 554 megabases of which 549 were assigned to six pseudomolecules that appear complete with telomeric repeats at their extremes and large centromeric regions with low gene density. The new *L. japonicus* Gifu reference genome and associated expression data represent valuable resources for legume functional and comparative genomics. Here, we provide a first example by showing that the symbiotic islands recently described in *Medicago truncatula* do not appear to be conserved in *L. japonicus*.

## 1. Introduction

The roots of most plants are colonized by mycorrhizal fungi. This symbiotic interaction is ancient, perhaps dating back to the origin of land plants, and many of its genetic components have been co-opted to allow symbiotic nitrogen fixation in legumes.^1^ Much of the overlapping genetic framework, as well as components specific to both types of symbioses, have been uncovered using the model legumes *Lotus japonicus* (Lotus) and *Medicago truncatula* (Medicago).^2^ Lotus is a perennial legume that has a short generation time, abundant flowers, and a small diploid genome with an estimated size of ∼500 Mb.^3^ In addition, Lotus is self-compatible and amenable to tissue culture and *Agrobacterium* transformation.^4^ It has been used very successfully for forward genetic studies, resulting in the first identification of a plant gene (*Nin*) required for nodulation,^5^ and the discovery of receptors for rhizobium Nod factors (NFR1 and NFR5)^6^ and exopolysaccharides (EPR3).^7^

Lotus is also interesting from a legume phylogenetic point of view, as it is a member of the Robinoid clade, which lacks other species with comprehensive genetic and genomic resources. The Robinoids are part of the larger Hologalegina clade, which also includes the IRLC clade that comprises Medicago and important crops such as pea (*Pisum sativum*), chickpea (*Cicer arietinum*), alfalfa (*Medicago sativa*), and white clover (*Trifolium repens*).^8^ The Hologalegina clade is sister to the Indigoferoid/Milettioid clade that includes soybean (*Glycine max*), common bean (*Phaseolus vulgaris*), pigeon pea (*Cajanus cajan*), and cowpea (*Vigna unguiculata*).^8^ All these species engage in symbiotic nitrogen fixation, but their root nodule morphology differs. The Indigoferoid/Milettioid species soybean and common bean and the Robinoid species Lotus produce round, determinate nodules, while the IRLC legumes instead form elongated, indeterminate nodules with persistent meristems.^9^High-quality genetic and genomic Lotus resources will thus nicely complement those of other well-characterized legume species, facilitating functional, comparative, and phylo-genomic studies of symbiotic nitrogen fixation, arbuscular mycorrhization, and other legume traits of interest.

The genetic resources already available for Lotus include sequenced natural accessions^10^ and recombinant inbred lines (RILs),^11^^,^^12^ as well as extensive populations of TILLING lines^13^ and *LORE1* insertion mutants.^14^ In addition, large volumes of Lotus expression and *LORE1* data have been integrated in the online portal *Lotus* Base^15^ (https://lotus.au.dk). Two Lotus accessions, MG-20 and Gifu B-129 (Gifu), have been especially frequently used.^16^ So far, genome sequencing efforts have focused exclusively on MG-20, resulting in the release of version 1.0, 2.5, and 3.0 MG-20 assemblies^17^ (https://www.kazusa.or.jp/lotus/ and https://lotus.au.dk/). MG-20 version 3.0 is a hybrid assembly based on Sanger and Illumina data that comprises 132 scaffolds covering 232 Mb aligned to the six Lotus chromosomes and an additional 162 Mb of sequence in 23,572 unanchored contigs. This MG-20 assembly has proved very useful for genetic mapping and for genome-wide transcriptome, methylation, and insertion mutant analyses,^7^^,^^14^^,^^18^^,^^19^ but it remains incomplete. Gifu originates from central Japan and is closely related to most of the sequenced accessions,^10^ whereas MG-20 is an atypical Lotus accession that originates from Miyakojima Island in the far south of Japan close to Taiwan. Considering also that the *LORE1* insertion mutant collection^14^ was generated in the Gifu background, a high-quality Lotus Gifu reference genome would not only facilitate comparative genomics studies, but also serve to underpin improvement of functional genomics and intraspecific diversity resources in Lotus.

Here, we present a high-quality Lotus Gifu reference assembly constructed based on ∼100× PacBio read coverage and scaffolded using Hi-C and high-resolution genetic map data. We use this high-quality assembly to explore the positional clustering of putative orthologs of Medicago lncRNAs and compare nodule-regulated gene clusters between Lotus and Medicago. Conserved gene regulation was found for root and nodule samples, but evidence supporting conservation of the symbiotic islands discovered in Medicago did not emerge.

## 2. Materials and methods

### 2.1. PacBio data generation and assembly

Lotus Gifu high-molecular weight DNA was extracted as described^20^ and sent to Earlham Institute and Takara Bio Inc. for PacBio sequencing. A total of 11.8 million reads with an average length of 8 kb were generated. The PacBio reads were assembled using Canu (version 1.3)^21^ with the parameters: corOutCoverage = 100, errorRate = 0.015, corMhapSensitivity = normal, corMaxEvidenceErate = 0.15, oeaMemory  = 15, cnsMemory = 40. The assembled contigs were then polished using PacificBiosciences’ GenomicConsensus package using Quiver (https://github.com/PacificBiosciences/GenomicConsensus).

### 2.2. Constructing genetic maps based on data from two RIL populations

Paired-end reads from RILs of Gifu × *Lotus burttii* and Gifu × MG-20, as well as those from their respective parental lines (Lotus Gifu, Lotus MG-20, and *L. burttii*), were mapped to the polished assembly using BWA-MEM.^22^ Picard (http://broadinstitute.github.io/picard/) was used to dedupe the generated BAM files, followed by variant calling using mpileup provided by SAMtools.^23^ The resulting VCF files were filtered based on the following criteria: (1) minimum quality of 30, (2) minimum depth of 50, (3) must be biallelic, and (4) cannot contain missing genotypes. To improve the quality of the genetic map, further filtering was performed using a Python script to select solely for single-nucleotide polymorphisms (SNPs) that are homozygous in the Gifu parent and homozygous alternative in the second RIL parent (MG-20 or *L. burttii*). To generate a consensus genotype call pattern for each contig across each RIL population (Gifu × *L. burttii* and Gifu × MG-20), the most commonly occurring genotype across all positions was selected.

### 2.3. Assembly scaffolding based on genetic maps and Hi-C data

Gifu leaf tissue was sent to Phase Genomics (https://phasegenomics.com), where Hi-C sequencing was carried out and a draft proximity-based (Proximo) scaffolding generated. Chromatin conformation capture data were generated using a Phase Genomics (Seattle, WA) Proximo Hi-C.^24^ Intact cells from two samples were crosslinked using a formaldehyde solution, digested using the *Sau*3AI restriction enzyme, and proximity ligated with biotinylated nucleotides to create chimeric molecules composed of fragments from different regions of the genome that were physically proximal *in vivo*, but not necessarily genomically proximal. Molecules were pulled down with streptavidin beads and processed into an Illumina-compatible sequencing library. Sequencing was performed on an Illumina NextSeq 500, generating a total of 175,495,827 PE150 read pairs. Reads were aligned to the draft PacBio assembly scaffoldSeq.fasta using bwa mem with the -5 option.^22^ Alignments were then filtered with SAMtools^23^ using the -F 2316 filtering flag.

Phase Genomics’ Proximo Hi-C genome scaffolding platform was used to create chromosome-scale scaffolds from the draft assembly in a method similar to that described by Bickhart et al.^25^ As in the LACHESIS method,^26^ this process computes a contact frequency matrix from the aligned Hi-C read pairs, normalized by the number of *Sau*3AI restriction sites (GATC) on each contig, and constructs scaffolds in such a way as to optimize expected contact frequency and other statistical patterns in Hi-C data. Approximately 88,000 separate Proximo runs were performed to optimize the number of scaffolds and scaffold construction in order to make the scaffolds as concordant with the observed Hi-C data as possible. This process resulted in a set of six chromosome-scale scaffolds containing 549 Mb of sequence (>99% of the draft assembly). Chimeric contigs were identified based on genetic map, Hi-C, and PacBio coverage data and split. The initial scaffolding was then iteratively improved using genetic map data followed by re-running Proximo scaffolding until genetic map and proximity-based scaffolding results converged.

### 2.4. Genome annotation

The annotation of the Lotus Gifu genome was performed using evidence from transcriptome data as well as homology information from related species. For the homology-based annotation, available *Arabidopsis thaliana* (Araport11), *Glycine max* (version 2.1), and Medicago (MtrunA17r5.0-ANR) protein sequences were combined. These protein sequences were mapped to the Lotus Gifu reference genome sequence using the splice-aware alignment tool GenomeThreader^27^ (version 1.6.6; with the arguments -startcodon -finalstopcodon -species rice -gcmincoverage 70 -prseedlength 7 -prhdist 4). In the expression data-based step, multiple RNA-seq datasets (SRP127678, SRP105404, DRP000629, PRJNA622801) were used as evidence for the genome-guided prediction of gene structures. Therefore, reads from RNA-seq datasets were mapped to the genome using Hisat2 (version 2.1, parameter –dta)^28^ and subsequently assembled into transcript sequences with Stringtie (version 1.2.3, parameters -m 150 -t -f 0.3).^29^ Next, Transdecoder (version 3.0.0) (https://github.com/TransDecoder/TransDecoder) was used to identify potential open reading frames and predict protein sequences. Using BLASTP (ncbi-blast-2.3.0+, parameters -max_target_seqs 1 -evalue 1e-05),^30^ the predicted protein sequences were compared against a protein reference database (UniProt Magnoliophyta, reviewed/Swiss-Prot) and used hmmscan (version 3.1b2)^31^ to identify conserved protein family domains for all proteins. BLAST and hmmscan results were then used by Transdecoder-predict and the best translations per transcript sequence was selected. Finally, results from the two gene prediction approaches were combined and redundant protein sequences were removed. Additionally, some symbiosis genes were manually curated (Supplementary Table S6).

In order to classify gene models into complete and functional genes, non-coding transcripts, pseudogenes, and transposable elements, a confidence classification protocol was applied. Candidate protein sequences were compared against the following three databases using BLAST: PTREP, a manually curated database of hypothetical proteins that contains deduced protein sequences, from which frameshifts have mostly been removed (http://botserv2.uzh.ch/kelldata/trep-db/index.html); Fab, a database with annotated proteins from the legumes *Glycine max* and Medicago; and UniMag, a database of validated proteins from the Magnoliophyta. UniMag protein sequences were downloaded from UniProt and further filtered for complete sequences with start and stop codons. Best hits were selected for each predicted protein to each of the three databases. Only hits with an *E*-value below 10e-10 were considered. Furthermore, only hits with subject coverage above 80% were considered significant and protein sequences were further classified into high and low confidence. High-confidence (HC) protein sequences are complete and have a subject and query coverage above the threshold in the UniMag database (HC1) or no blast hit in UniMag but in Fab and not PTREP (HC2). While a low-confidence (LC) protein sequence is not complete and has a hit in the UniMag or Fab database but not in PTREP (LC1), or no hit in UniMag and Fab and PTREP but the protein sequence is complete. Functional annotation of transcripts as well as the assignment of GO terms was performed using the tool ‘Automatic assignment of Human Readable Descriptions – AHRD’. AHRD performs BLASTP search against Swiss-Prot, The Arabidopsis Information Resource (TAIR), and TrEMBL databases to perform functional annotation based on homology to other known proteins and integrates domain search results from InterProScan as well as gene ontology (GO) terms.^32^ Repeats were annotated using RepeatMasker^33^ version 3.3 with a custom Fabaceae-library in sensitive mode. Non-coding RNAs were predicted using tRNAscan-SE (version 1.3.1),^34^ RNAmmer (version 1.2),^35^ and Infernal (version 1.1.2)^36^ with default parameters. The results were merged subsequently.

### 2.5. Expression atlas

Raw Lotus Gifu RNA-seq reads were obtained from either the Sequence Read Archive (SRA) for the listed accessions or generated in this study (Supplementary Table S1). For data in this study, 3-day old Lotus Gifu seedlings were transferred to filter paper covered agar (1.4% agar noble) slants. Roots were treated with *M. loti* R7A, 6-Benzylaminopurine (BA) (1 µM) or mock and a 1-cm segment of root tissue corresponding to the zone of emerging root hairs at time of treatment was harvested. For nodule tissue, whole nodules were harvested. Libraries were constructed and sequenced by Novogene (Hong Kong) using PE-150bp reads on the Illumina NovaSeq 6000 instrument. A decoy-aware index was built for Gifu transcripts using default Salmon parameters and reads were quantified using the –validateMappings flag^37^ (Salmon version 0.14.1). A normalised expression atlas across all conditions was constructed using the R-package DESeq2 version 1.20^38^ after summarizing gene level abundance using the R-package tximport (version 1.8.0). Normalized count data obtained from DESeq2 are available in the *Lotus* Base expression atlas (https://lotus.au.dk/expat/).^15^

### 2.6. Analysis of symbiotic islands

Medicago A17 proteins associated with symbiotic islands as defined by Pecrix et al.,^39^ were blasted against Lotus Gifu proteins annotated in the present assembly, and the best hit was extracted. It was then determined if there was microsynteny between the Medicago A17 genes in the symbiotic island and the best Lotus Gifu matches (Supplementary File S5). Medicago A17 RNA-seq data (Supplementary Table S2) was trimmed using trimmomatic (10.1093/bioinformatics/btu170), trimmed reads were mapped to the Medicago A17 version 5 reference sequence (MtrunA17r5.0) using the splice aware STAR aligner (version 2.5.1a).^40^ A read was allowed to map in at most 10 locations (–outFilterMultimapNmax 10) with a maximum of 4% mismatches (–outFilterMismatchNoverLmax 0.04) and all non–canonical intron motifs were filtered out (–outFilterIntronMotifs RemoveNoncanonicalUnannotated). In order to obtain non-unique gene-level counts from the mapping files, HTSeq (version 0.9.1)^41^ with the ‘nonunique all’-method was used. Normalization of read counts was performed by library sequence depth using the R-package DESeq2 (version 1.23.3).^38^

Log expression ratios of 10 days post inoculation (dpi) nodule samples versus non-inoculated root samples were calculated for Lotus Gifu and Medicago A17 and Pearson correlation coefficients were calculated (Supplementary Tables S1 and S2). For calculation of Pearson correlation coefficients, all Medicago A17 RNA-seq samples listed in Supplementary Table S2 were used, while only Lotus Gifu root and nodule samples were used (Supplementary Table S1). When analyzing the largest possible set of genes (Fig. 3B), all Medicago A17 genes with a match to a Lotus Gifu gene anywhere in the genome were included along with one Lotus Gifu match per Medicago A17 gene, allowing many copies of the same Lotus Gifu gene. For analysis of unique Lotus genes, only a single Medicago A17 gene was included per Lotus Gifu match within the microsyntenic region and islands with less than three Lotus Gifu microsyntenic hits were not considered (Fig. 3C). All statistical analyses were carried out using R version 3.4.3. The scripts used for analysis are freely available from GitHub (https://github.com/stiguandersen/LotjaGifuGenome).

### 2.7. Data availability

Sequencing data are available from SRA. PacBio data used for genome assembly and Hi-C data from Phase Genomics used for construction of proximity map (PRJNA498060); Illumina paired-end data from RIL resequencing used for genetic map construction (PRJNA498068); *L. burttii* genomic DNA reads (PRJNA635235); RNA-seq data used for annotation (PRJNA622801); RNA-seq expression atlas data (PRJNA622396). Assembly pseudomolecules are available from the NCBI nucleotide repository with accession numbers AP022629–AP022637. Pseudomolecule sequences and genome annotation information are also found in Supplementary Files S2 and S3 and are available for browsing and download at *Lotus* Base (https://lotus.au.dk) and LegumeBase (https://www.legumebase.brc.miyazaki-u.ac.jp) and for synteny comparisons at CoGe (https://genomevolution.org/coge/GenomeInfo.pl?gid=58121).

## 3. Results and data description

### 3.1 A chromosome-scale Lotus Gifu assembly including telo- and centromeric repeats

We generated a total of 11.8 million PacBio RSII reads, which we assembled using Canu^21^ into 1,686 contigs with an N50 of 807 kb and a total length of 554 Mb (Table 1). We first scaffolded the contigs using 175 million Proximo Hi-C reads (Phase genomics). To validate the scaffolding, we mapped whole genome re-sequencing data from two RIL populations^12^ to the PacBio contigs. The vast majority of the assembly, 99.5%, was contained within contigs that had at least one polymorphic SNP marker, leaving only 2.5 Mb of sequence on markerless contigs (Table 1). We compared the Hi-C scaffolding results to the genetic maps generated based on the RIL data (Supplementary File S1) and moved contigs according to genetic linkage. We then repeated the scaffolding until the Proximo Hi-C results were concordant with the genetic maps and the contigs were arranged in six pseudomolecules corresponding to the six Lotus chromosomes (Supplementary File S2). The total length of the assembly was close to the expected genome size of ∼500 Mb (Table 1), and we found canonical telomeric repeats at the ends of all pseudomolecules, except for the bottom of chromosome 3, indicating a high completeness of the assembly. The 2.5 Mb of unanchored contigs placed on chr0 contained a substantial amount of pericentromeric repeats.

**Table 1:** Assembly and genetic map statistics

Dataset	Contig count	Total length (bp)	N50 (bp)	L50
Assembly	1,686	554,078,227	807,552	187
Containing ≥1 SNP	1,538 (91.2%)	551,215,263 (99.5%)	823,414	185
Exclusively Gifu × *L. burttii*	105 (6.3%)	3,270,218 (0.6%)	35,963	27
Exclusively Gifu × MG-20	124 (7.4%)	5,010,333 (0.9%)	51,370	29
Contains SNPs from both	1,309 (77.6%)	542,934,712 (97.9%)	835,713	180
Does not contain any SNPs	148 (8.8%)	2,862,964 (0.5%)	23,531	46

N50: at least 50% of the total length is contained within contigs of size N50 or longer. L50: at least 50% of the total length is contained within L50 number of contigs.

**Figure 1:**
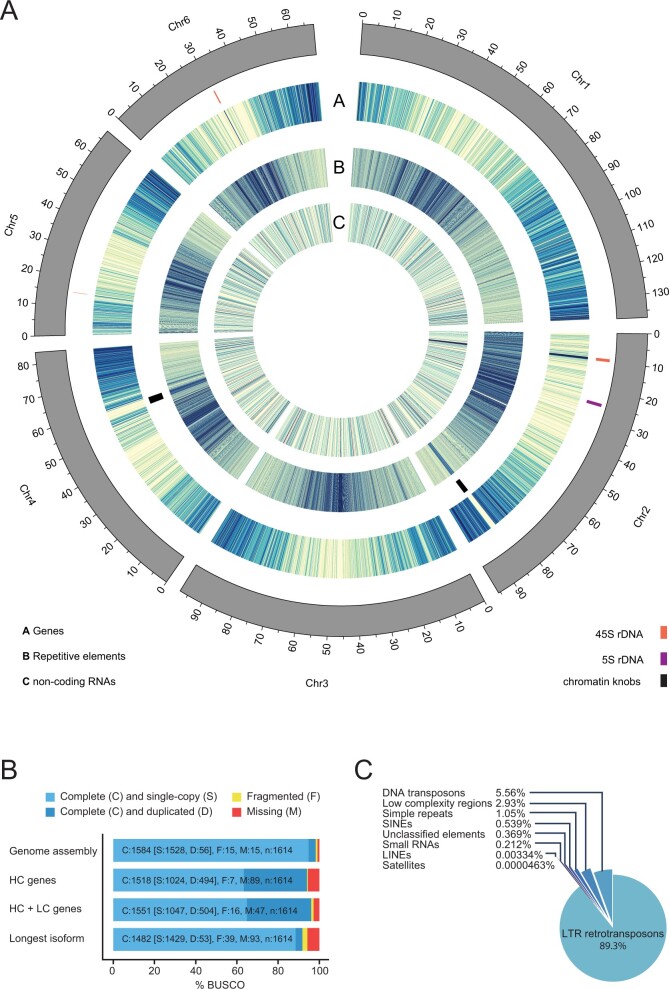
(A) Circos diagram displaying heatmaps of the numbers of genes and ncRNAs (100 Mb bins) and bases covered by repetitive elements (10 Mb bins) in the Lotus Gifu genome. (B) BUSCO version 4 scores of the Lotus Gifu assembly (98.2%), the HC gene set (94%), the high- and LC gene set (96.1%) and of only the longest transcript of each gene (91.8%) from the joint HC and LC gene set. Lineage used: embryophyta_odb10. (C) Distribution of repetitive elements in the Lotus Gifu genome.

Regarding the highly repetitive sequences, three 45S rDNA clusters and a 5S rDNA gene cluster were anchored on chromosomes 2, 5, and 6, and on chromosome 2, respectively, consistent with FISH data (Fig. 1A).^42^ In addition to the regions with a high density of repetitive sequences, corresponding to the pericentromeric regions of each chromosome, small regions with high densities of repetitive sequences were identified within the gene rich regions at the bottom arm of chromosomes 2 and 4 (Fig. 1A). The location of these regions corresponded to the positions of chromosome knobs reported in the previous cytological analyses.^42^^,^^43^ These regions with highly dense repetitive sequences tend to be composed of contigs with short length, and thus a significant number of the sequence gaps (389 out of 1,555) were found in these regions. Despite the relatively high frequency of sequence gaps in these repetitive regions, the Hi-C reads provided sufficient physical linking information to allow scaffolding.

### 3.2. Genome annotation

Based on evidence from expression data as well as homology information from related species, 30,243 genes were annotated, 21,778 of which represent HC gene models (Table 2, Supplementary File S3). Using the embryophyta_odb10 lineage 1,584 out of 1,614 (98.2%) complete BUSCO v4 orthologs^44^ were found in the genome assembly and 1,551 (96.1%), were identified within the annotated gene set (Fig. 1B). The HC gene set had a BUSCO score of 94%. Using AHRD,^32^ we could assign functional annotations to 29,429 genes (97%). Of these, 70.53% fulfilled all three AHRD quality criteria, 16.85% fulfilled two and 11.8% fulfilled one criterion. We then annotated non-coding RNAs, identifying 2,933 in total that comprised 128 micro RNAs, 851 snoRNAs, 88 tRNAs, 795 rRNAs, and others. In total, gene models covered 156,379,918 bases and coding exons covered 60,649,299 bases of the genome assembly.

**Table 2:** Genome annotation statistics

	Lotus Gifu version 1.2 HC + LC	Lotus Gifu version 1.2 HC	Medicago A17 version 4	Medicago A17 version 4 HC	Medicago A17 version 5	*Glycine max* Williams 82 version 2.1
Number of genes	30,243	21,778	50,444	31,451	51,316	52,872
Number of coding genes	29,554	21,778	50,444	31,451	44,623	52,872
Number of mRNAs	49,868	37,994	57,585	38,175	44,623	86,256
Number of exons	306,545	264,198	267,394	397,385	189,379	560,910
Number of CDSs	262,442	236,845	257,792	376,276	174,461	516,059
Average CDS lengths (bp)	1,216.2	1,385.4	1,038.4	1,272.4	1,017.7	1,350.7
Average exon lengths (bp)	417.54	373.77	282.58	261.92	360.25	312.48
Average intron lengths (bp)	527.12	513.71	444.41	438.41	476.57	519.19
Average transcripts per gene	1.65	1.74	1.14	1.54	1	1.63
Average exons per transcript	6.15	6.95	4.64	6.75	4.19	6.5
Average CDS exons per transcript	5.23	6.23	4.48	6.39	3.91	5.98

HC, high-confidence gene models; LC, low-confidence gene models.

Repetitive elements made up 260,312,827 bases (46.96%) of the genome. Of these, long-terminal repeat retrotransposons accounted for most of the repeat content of the genome (42.51%), followed by DNA transposons and low complexity regions (Fig. 1C). Chromosomes 1, 3, 4, 5, and 6 showed centrally located pericentromeric regions rich in repetitive elements flanked by gene-rich regions (Fig. 1A). In contrast, the centromere of chromosome 2 appeared to be distally located near the top of the chromosome, which also carried a large cluster of rRNA genes (Fig. 1A).

### 3.3. RNA-seq-based expression atlas

**Figure 2:**
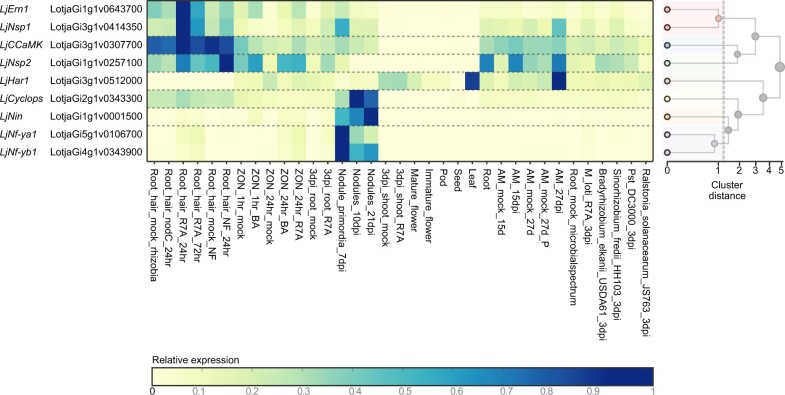
Expression profiles of known symbiosis genes. Expression values from the Lotus Gifu RNA-seq expression atlas are shown for the indicated genes. A full description of the conditions included is shown in Supplementary Table S1. The heatmap was generated from https://lotus.au.dk/expat/ using the normalize by row function.

To produce a gene expression atlas, publicly available and new RNA-seq data from Lotus Gifu was obtained for 35 conditions across different tissues, symbiotic, and pathogenic interactions (Supplementary Table S1). The conditions available include root hair, nodule primordia, and nodules obtained after inoculation with *Mesorhizobium loti* R7A and root interactions with microbes across a symbiont-pathogen spectrum;^18^ root and shoot tissues 3 days after roots were inoculated with *M. loti*^45^; root symbiotic susceptible zone treated with cytokinin (1 µM BA) or *M. loti* R7A (this study); roots inoculated with the arbuscular mycorrhizal fungus (AMF), *Glomus intraradices*^46^; root, leaf, immature flowers, mature flowers, pods, and seeds (SRA ID: PRJDB2436). Gene-level quantification of the data was normalized across conditions (Supplementary File S4) and is made available through *Lotus* Base (https://lotus.au.dk/expat/) to provide a readily accessible expression viewer. Well-described nodulation genes showed the expected expression patterns across the conditions represented in the expression atlas (Fig. 2).

### 3.4 Symbiotic islands are not generally conserved between Lotus and Medicago

Recently, ‘symbiotic islands’ representing clusters of genes that showed co-regulated, symbiosis-related expression profiles were identified in Medicago A17.^39^ Interestingly, these clusters were rich in long non-coding (lnc) RNAs, and it was proposed that the lncRNAs may be involved in regulating symbiosis-related gene expression. To investigate if the Medicago symbiotic islands were conserved in Lotus, we extracted the best Lotus Gifu BLAST hits against the Medicago A17 genes reported to reside within symbiotic islands (Supplementary File S5). Protein coding genes were generally well conserved and showed high levels of microsynteny, regardless of whether or not they were present in gene islands that showed symbiosis-related differential expression (Table 3). Out of 760 islands, 266 had at least three distinct Lotus Gifu hits in microsyntenic regions, and the region with the largest overlap comprised 12 hits. In contrast, most Medicago A17 lncRNAs had no putative orthologs in the Lotus Gifu genome, and, when identified, they were often not found within the designated microsyntenic region (Table 3). Across all 760 investigated islands, a total of six had two lncRNA hits to the Lotus Gifu microsyntenic region, and no island had more than two.

**Table 3:** Conservation of symbiotic islands between Lotus and Medicago

Island type	NRU	NRD	NRN	NDA	NDD	NDN
Islands	270	89	84	49	211	57
Mt genes	2559	712	628	377	1680	429
Mt genes with Lj hits	1040	550	516	298	506	322
Corresponding Lj genes	770	358	456	261	396	275
Lj genes with micro-synteny	446	228	320	190	215	166
Mt lncRNAs	302	40	17	25	228	18
Mt lncRNAs with Lj hit	47	17	9	13	31	6
Mt lncRNAs with Lj hit in micro-syntenic region	17	3	5	9	8	5
Conservation rate (%)	40.6%	77.2%	82.2%	79.0%	30.1%	75.1%
Duplication rate	1.35	1.54	1.13	1.14	1.28	1.17
Ratio of genes with micro-synteny	57.9%	63.7%	70.2%	72.8%	54.3%	60.4%
Islands with Lj hits in more than half of the genes	101 (37%)	80 (90%)	82 (98%)	44 (90%)	36 (17%)	49 (86%)

Mt, Medicago A17; Lj, Lotus Gifu; NRU, nodule versus root upregulated; NRD, nodule versus root downregulated; NRN, nodule versus root not regulated; NDA, nodule development apical zone; NDD, nodule development differentiation zone; NDN, nodule development not regulated.

**Figure 3:**
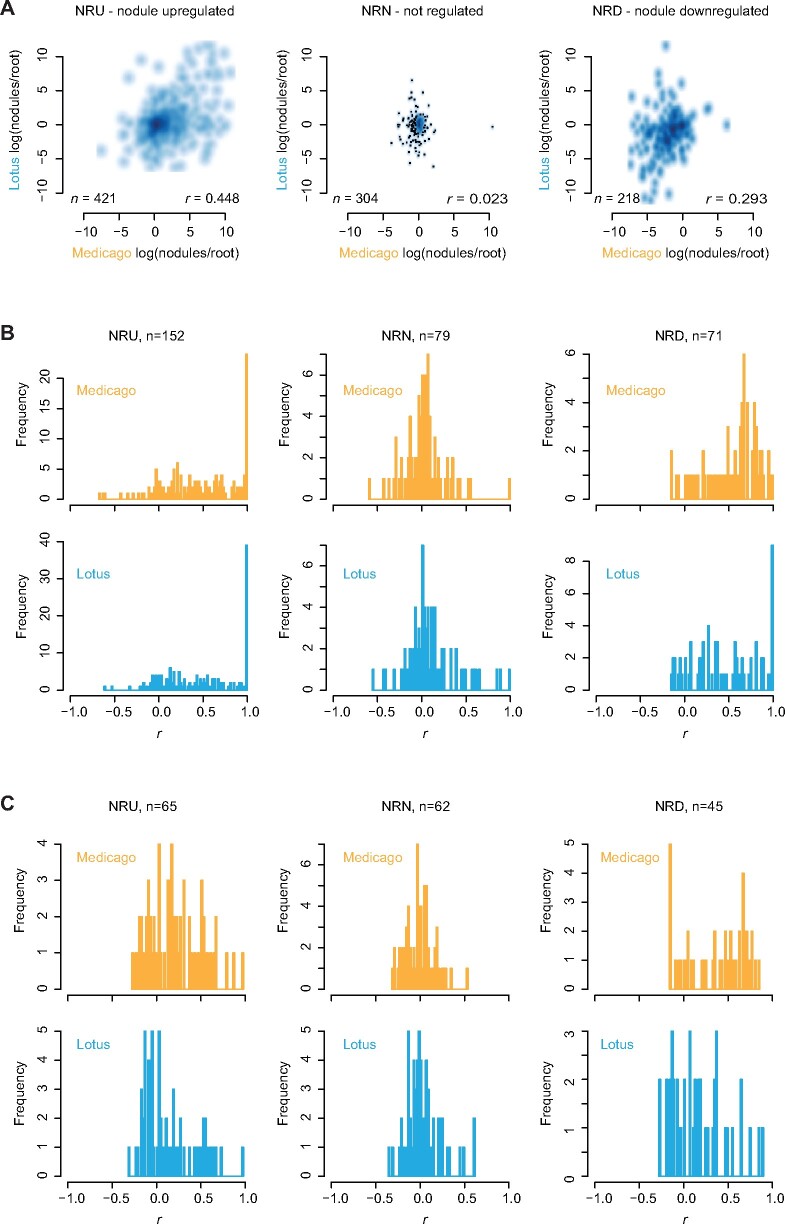
Symbiotic island gene expression. (A) Log(nodule/root) expression ratios for genes in Medicago A17 symbiotic islands and their best Lotus Gifu BLAST matches. *n*: gene count. *r*: Pearson correlation coefficient for the Lotus Gifu and Medicago A17 log(nodule/root) ratios. (B–C) Histograms of Pearson correlation coefficients for symbiotic islands. The Pearson correlation coefficient for each island is an average of the coefficients resulting from pairwise comparisons of the gene expression profiles of all genes residing within that island. *n*: symbiotic island count. (B) All genes in Medicago A17 symbiotic islands with a putative Lotus Gifu homolog with expression data. Multiple copies of the same Lotus Gifu gene are allowed. (C) Only one Lotus Gifu copy and one corresponding Medicago A17 gene is included in the analysis and it is further required that each Lotus Gifu island contains at least three members. Lotus: expression data from Lotus Gifu. Medicago: expression data from Medicago A17.

The limited conservation and lack of positional clustering make it unlikely that putative orthologs of Medicago lncRNAs are generally part of symbiotic islands in Lotus. Instead, we looked further into the protein coding genes to determine if their organization into symbiotic islands could be conserved. All 760 islands contain at least one protein coding gene. Out of these, we examined the 443 islands associated with nodule-regulated genes designated ‘Nodule upregulated (NRU)’, ‘Nodule downregulated (NRD)’, and ‘Nodule non-regulated (NRN)’. First, we investigated the level of expression conservation by comparing the expression of Medicago A17 genes in symbiotic islands and their Lotus Gifu syntenic homologs in root and 10 dpi nodule samples (Supplementary Tables S1 and S2 and Supplementary Files S4 and S6). The genes associated with Medicago A17 NRU islands showed strongly correlated expression responses in Lotus Gifu and Medicago A17, NRDgenes showed a less pronounced correlation, while there was no correlation for the NRN genes (Fig. 3A).

To quantify the level of co-regulation within putative symbiotic islands, we calculated the average Pearson correlation coefficients for each island based on the gene expression data from root and nodule samples (Supplementary Tables S1 and S2). First, we included all genes in Medicago symbiotic islands that had a Lotus BLAST match anywhere in the genome along with their best Lotus match. If a Lotus gene was the best match for multiple Medicago genes, it was included multiple times in the analysis. Especially for the NRU islands, this resulted in a very pronounced skew towards high correlation coefficients as compared to the NRN islands, and this was true both for Lotus Gifu and Medicago A17 (Fig. 3B).

We then repeated the analysis including only Medicago A17–Lotus Gifu syntenic homolog pairs from islands with at least three unique Lotus Gifu genes. That is, if multiple Medicago A17 genes matched the same Lotus Gifu gene, only a single Medicago A17 gene was retained and each unique Lotus Gifu gene was only included once per island. This resulted in a marked reduction in the number of islands and the large peak of near perfect correlation coefficients for NRU islands disappeared for both Lotus Gifu and Medicago A17 (Fig. 3C). Since there was no longer a major difference between the root/nodule-based correlation coefficients between the nodule-regulated NRU and NRD islands and the NRN controls, it appears that local gene amplification in MedicagoA17 is a major cause of the symbiotic island signal. This is consistent with an overall high ratio of Medicago A17 to Lotus Gifu genes in symbiotic islands (Table 3). Symbiotic islands are thus not generally conserved between Lotus and Medicago and are not general features of legume genomes. However, we did find a few examples of gene clusters that showed conserved co-regulation for root and nodule samples (Supplementary Tables S3–S5). In Lotus Gifu, NRU island SRI_NDD0105, which had the second highest Lotus correlation coefficient (Supplementary Table S3), had three very similar copies of a nodulin gene, suggesting that local gene amplification also plays a role here. In contrast, the NRU island with the highest Lotus correlation coefficient (SRI_NRU0026) comprised three very different genes, perhaps warranting further investigation (Supplementary Table S3).

## 4. Conclusion and future perspectives

By applying long PacBio reads, the contiguity of the assembly was improved compared to the Lotus MG-20 version 3.0 assembly that was a hybrid assembly based on Sanger and Illumina sequences. Using Hi-C paired-end reads and high-density SNP marker information generated by re-sequencing of Lotus Gifu x *L. burttii* RILs, 1,584 contigs were anchored onto 6 chromosomes with 42 scaffolds, providing a high-quality and well-validated assembly. The number of scaffolds was a bit larger than that of the latest Medicago A17 sequence (Mt5.0) due to manual correction of Hi-C scaffolding errors based on the SNP marker information. Typical Hi-C scaffolding errors were identified in the distal regions of each pseudomolecule and at the border regions of chromosome knobs located on chromosomes 2 and 4, presumably due to an atypical three-dimensional chromosome conformation in those regions. A total of 30,243 high and LC gene models were annotated, which corresponds approximately to the number of HC gene models in the Medicago version 4 assembly (Table 2). The total number of annotated genes is higher for Medicago versions 4 and 5 than for the current Lotus Gifu assembly. However, the number of exons per transcript is markedly lower for the full Medicago A17gene sets than for the Lotus Gifu gene and Medicago A17 version 4 HC gene sets, suggesting that the differences in gene numbers are due to different stringencies in including small genes with few exons. As expected, the paleopolyploid soybean (*Glycine max*)^47^ has a higher number of annotated genes than Lotus but retains a similar exon per transcript ratio despite more than 50,000 annotated genes.

The availability of a high-quality Lotus Gifu assembly will facilitate further improvements of genetic and genomic Lotus resources. The *LORE1* mutant collection, which includes more than 130,000 insertion mutant lines, is in the Lotus Gifu genetic background, but was annotated based on the Lotus MG-20 version 3.0 assembly.^14^ Using the new Lotus Gifu sequence, the *LORE1* insertions can now be more accurately characterized. Likewise, Gifu is more closely related to the majority of the collection of natural Lotus accessions that was recently characterized,^10^ and the new reference assembly should allow an improved characterization of the genetic diversity. Here, we have mapped existing and new RNA-seq data to the Gifu assembly to provide a consistently normalized and updated Lotus gene expression atlas readily available through *Lotus* Base.^15^ The current atlas does not comprise as many samples as previously profiled using microarrays,^48^^,^^49^ but it is not limited by probe set selection and includes data on all annotated and expressed genes.

The new assembly and expression atlas also proved useful in interspecific comparisons, since the complete pseudomolecules allowed us to accurately assess synteny with Medicago to investigate the level of conservation of plant symbiotic islands. Interestingly, the recently identified Medicago symbiotic islands did not appear to be conserved in Lotus. This was most evident for the Medicago non-coding RNAs, for which we could find only very few matching sequences in Lotus despite the completeness of the assembly. It should be noted that many of the transcripts classified as lncRNAs in the Medicago study^39^ in fact encode peptides, most notably the large family of nodule cysteine-rich (NCR) peptides. The NCR peptides are characteristic of the Inverted Repeat Lacking Clade (IRLC) legume lineage and thus not found in Lotus.^50^ The same appears to be the case for the other transcripts in the non-coding class, indicating that non-coding and peptide-encoding genes have evolved rapidly and are not generally required for legume-rhizobium symbiosis across determinate and indeterminate nodulators. For the protein-coding genes in symbiotic islands, we found much higher levels of conservation and microsynteny, but most of the local co-regulation appeared to be related to tandem gene duplications in Medicago. Generally, Medicago seems to have experienced not only a rapid expansion of NCR peptide genes and lncRNAs involved in symbiosis, but also of protein-coding genes with symbiosis-related expression patterns, and our results clearly indicate that symbiotic islands are not general features of legume genomes.

The analysis of symbiotic islands represents only a first use case for the new high-quality Lotus Gifu genomic data, and we anticipate that it will be broadly used in genomics studies. The data will be included in comparative genomics websites such as Phytozome^51^ and Legume Information System^52^ and it is already available at CoGe (https://genomevolution.org/coge/GenomeInfo.pl?gid=58121).^53^ In addition, the high completeness of the assembly and the set of annotated genes makes the data well suited for phylogenomic studies that rely on precise genomic data for large-scale cross-species analyses.^54^

## Supplementary data

Supplementary data are available at *DNARES* online.

## Author contributions

Conceptualization, S.U.A., S.S. and K.F.X.M; validation, N.K., S.S., K.F.X.M and S.U.A.; formal analysis, N.K., T.M., D.R., T.Y.A., T.A., H.H., S.S. and SUA; investigation, N.S; resources, J.S., S.S., K.F.X.M., S.U.A.; data curation, N.K., S.S., K.F.X.M and S.U.A.; writing—original draft, S.U.A. and N.K.; writing—review and editing, S.U.A. and N.K. with input from all authors; visualization, N.K., S.U.A. and D.R.; supervision, S.U.A., S.S. and K.F.X.M.; project administration, J.S., S.S., K.F.X.M. and S.U.A.; funding acquisition, J.S., S.S., K.F.X.M. and S.U.A.

## Supplementary Material

dsaa015_Supplementary_Data
