# Supplementary material for: Insights into the evolution of symbiosis gene copy number and distribution from a chromosome-scale Lotus japonicus Gifu genome sequence
Source: DNA Res. 2020 Jul 13;27(3):dsaa015. doi: 10.1093/dnares/dsaa015 (PMC7508351; doi:10.1093/dnares/dsaa015)
Supplement: dsaa015_Supplementary_Data [file dsaa015_supplementary_data.zip › 20200629_Gifu_genome_suppl.docx]

**Supplemental information for Kamal et al.**

# Supplemental tables

| **Tissue** | **Sample name** | **Nod regulation** | **Correlation** | **Replicates** | **Reference** | **Bioproject ID** |
| --- | --- | --- | --- | --- | --- | --- |
| Root susceptible zone | ZON_1hr_mock |  |  | 3 | Current study | PRJNA622396 |
| Root susceptible zone | ZON_1hr_BA |  |  | 3 | Current study | PRJNA622396 |
| Root susceptible zone | ZON_24hr_mock |  |  | 3 | Current study | PRJNA622396 |
| Root susceptible zone | ZON_2 hr_BA |  |  | 3 | Current study | PRJNA622396 |
| Root susceptible zone | ZON_24hr_R7A |  |  | 3 | Current study | PRJNA622396 |
| Nodule | nodules_10dpi | x | x | 3 | Current study | PRJNA622396 |
| Root | 3dpi_root_mock | x | x | 3 | Munch et al., 2018 | PRJNA384655 |
| Root | 3dpi_root_R7A |  | x | 3 | Munch et al., 2018 | PRJNA384655 |
| Shoot | 3dpi_shoot_mock |  |  | 3 | Munch et al., 2018 | PRJNA384655 |
| Shoot | 3dpi_shoot_R7A |  |  | 3 | Munch et al., 2018 | PRJNA384655 |
| Flower | Mature_flower |  |  | 3 | NIBB | PRJDB2436 |
| Flower | Immature_flower |  |  | 3 | NIBB | PRJDB2436 |
| Pod | Pod |  |  | 3 | NIBB | PRJDB2436 |
| Seed | seed |  |  | 3 | NIBB | PRJDB2436 |
| Root | root |  | x | 3 | NIBB | PRJDB2436 |
| Leaf | leaf |  |  | 3 | NIBB | PRJDB2436 |
| Root | AM_15dpi |  |  | 3 | Handa et al., 2015 | PRJDB2819, PRJDB2576, PRJDB3212 |
| Root | AM_27dpi |  |  | 3 | Handa et al., 2015 | PRJDB2819, PRJDB2576, PRJDB3212 |
| Root | AM_mock_15d |  |  | 3 | Handa et al., 2015 | PRJDB2819, PRJDB2576, PRJDB3212 |
| Root | AM_mock_27d_ P |  |  | 3 | Handa et al., 2015 | PRJDB2819, PRJDB2576, PRJDB3212 |
| Root | AM_mock_27 |  |  | 3 | Handa et al., 2015 | PRJDB2819, PRJDB2576, PRJDB3212 |
| Root hair | root_hair_mock_rhizobia |  |  | 3 | Kelly et al., 2018 | PRJNA422278 |
| Root hair | root_hair_nodC_24h |  |  | 3 | Kelly et al., 2018 | PRJNA422278 |
| Root hair | root_hair_R7A_24hr |  |  | 3 | Kelly et al., 2018 | PRJNA422278 |
| Root hair | root_hair_R7A_72hr |  |  | 3 | Kelly et al., 2018 | PRJNA422278 |
| Root hair | root_hair_mock_NF |  |  | 3 | Kelly et al., 2018 | PRJNA422278 |
| Root hair | root_hair_NF_24hr |  |  | 3 | Kelly et al., 2018 | PRJNA422278 |
| Nodule | nodule_primordia_7_dpi |  | x | 3 | Kelly et al., 2018 | PRJNA422278 |
| Nodule | nodules_21dpi |  | x | 1 | Kelly et al., 2018 | PRJNA422278 |
| Root | Root_mock_microbialspectrum |  | x | 2 | Kelly et al., 2018 | PRJNA422278 |
| Root | M_loti_R7A_3dpi |  | x | 2 | Kelly et al., 2018 | PRJNA422278 |
| Root | Bradyrhizobium_elkanii_USDA61_3dpi | |  | 2 | Kelly et al., 2018 | PRJNA422278 |
| Root | Sinorhizobium_fredii_HH103_3dpi |  |  | 2 | Kelly et al., 2018 | PRJNA422278 |
| Root | Pseudomonas_syringae_pv_tomato_DC3000_3dpi |  |  | 2 | Kelly et al., 2018 | PRJNA422278 |
| Root | Ralstonia_solanacearum_JS763_3dpi |  |  | 2 | Kelly et al., 2018 | PRJNA422278 |

**Supplemental table 1.** Lotus Gifu RNA-seq samples. Nod regulation: Samples used for calculating expression differences between roots and nodules. Correlation: Samples used for calculating Pearson correlation coefficients for gene expression co-regulation in symbiotic islands. dpi: days post inoculation. NIBB: National Institute for Basic Biology, Japan.

| **SRA Sample ID** | **Sample Name** | **Tissue** | **Treatment** |
| --- | --- | --- | --- |
| SRR5740859 | MtNod0dpi_1 | Roots | Non-inoculated |
| SRR5740858 | MtNod0dpi_2 | Roots | Non-inoculated |
| SRR5740868 | MtNod0dpi_3 | Roots | Non-inoculated |
| SRR5740875 | MtNod4dpi_1 | Roots | 4 days post inoculation with *S. meliloti* 1021 |
| SRR5740878 | MtNod4dpi_2 | Roots | 4 days post inoculation with *S. meliloti* 1021 |
| SRR5740877 | MtNod4dpi_3 | Roots | 4 days post inoculation with *S. meliloti* 1021 |
| SRR5740870 | MtNod10dpi_1 | Nodules | 10 days post inoculation with *S. meliloti* 1021 |
| SRR5740864 | MtNod10dpi_2 | Nodules | 10 days post inoculation with *S. meliloti* 1021 |
| SRR5740861 | MtNod10dpi_3 | Nodules | 10 days post inoculation with *S. meliloti* 1021 |
| SRR5740862 | MtNod14dpi_1 | Nodules | 14 days post inoculation with *S. meliloti* 1021 |
| SRR5740866 | MtNod14dpi_2 | Nodules | 14 days post inoculation with *S. meliloti* 1021 |
| SRR5740869 | MtNod14dpi_3 | Nodules | 14 days post inoculation with *S. meliloti* 1021 |
| SRR5740860 | MtNod14dpi_12h_1 | Nodules | 14 days post inoculation with *S. meliloti* 1021, 12h nitrogen treatment |
| SRR5740871 | MtNod14dpi_12h_2 | Nodules | 14 days post inoculation with *S. meliloti* 1021, 12h nitrogen treatment |
| SRR5740874 | MtNod14dpi_12h_3 | Nodules | 14 days post inoculation with *S. meliloti* 1021, 12h nitrogen treatment |
| SRR5740873 | MtNod14dpi_48h_1 | Nodules | 14 days post inoculation with *S. meliloti* 1021, 48h nitrogen treatment |
| SRR5740872 | MtNod14dpi_48h_2 | Nodules | 14 days post inoculation with *S. meliloti* 1021, 48h nitrogen treatment |
| SRR5740876 | MtNod14dpi_48h_3 | Nodules | 14 days post inoculation with S. meliloti 1021, 48h nitrogen treatment |
| SRR5740867 | Mt4wkNod_1 | Nodules | 4 weeks post inoculation with *S. meliloti* 1021 |
| SRR5740865 | Mt4wkNod_2 | Nodules | 4 weeks post inoculation with *S. meliloti* 1021 |
| SRR5740863 | Mt4wkNod_3 | Nodules | 4 weeks post inoculation with *S. meliloti* 1021 |

**Supplemental table 2.** Medicago A17 RNA-seq data. SRA: Sequence read archive (<https://www.ncbi.nlm.nih.gov/sra>).

| **NRU islands** | ***r* Medicago** | ***r* Lotus** | **Island size** | **log(nodules/root) Lotus** | **log(root/nodules) Medicago** |
| --- | --- | --- | --- | --- | --- |
| SRI_NRU0026 | 0.53 | 0.97 | 3 | 11.37 | 7.49 |
| SRI_NRU0105 | 0.12 | 0.72 | 3 | -2.28 | 1.73 |
| SRI_NRU0009 | -0.17 | 0.67 | 3 | -2.80 | -0.96 |
| SRI_NRU0016 | 0.52 | 0.66 | 3 | 3.73 | 3.33 |
| SRI_NRU0032 | -0.07 | 0.61 | 3 | -1.54 | 1.93 |
| SRI_NRU0093 | 0.16 | 0.57 | 4 | 1.34 | 2.70 |
| SRI_NRU0209 | 0.26 | 0.55 | 3 | 4.25 | 0.98 |
| SRI_NRU0103 | 0.25 | 0.54 | 6 | 2.81 | 1.55 |
| SRI_NRU0059 | 0.52 | 0.53 | 3 | 6.15 | 3.31 |
| SRI_NRU0005 | 0.04 | 0.52 | 6 | -1.07 | 0.36 |
| SRI_NRU0170 | 0.60 | 0.52 | 3 | -0.91 | 2.30 |
| SRI_NRU0143 | 0.31 | 0.49 | 3 | 0.55 | 0.80 |
| SRI_NRU0246 | -0.11 | 0.47 | 3 | -1.13 | -0.13 |
| SRI_NRU0139 | 0.23 | 0.44 | 3 | -0.40 | 0.12 |
| SRI_NRU0156 | 0.32 | 0.43 | 4 | -1.03 | 3.05 |
| SRI_NRU0089 | 0.21 | 0.38 | 5 | 1.20 | 0.69 |
| SRI_NRU0207 | 0.66 | 0.31 | 4 | 2.81 | 3.96 |
| SRI_NRU0058 | 0.96 | 0.28 | 3 | 3.29 | 3.50 |
| SRI_NRU0019 | 0.66 | 0.27 | 3 | -0.81 | -2.07 |
| SRI_NRU0003 | 0.05 | 0.25 | 3 | -2.41 | 1.09 |

**Supplemental table 3.** Top correlated NRU islands. The table is sorted by the Lotus correlation coefficient (*r*). *r*: average Pearson correlation coefficient for pairwise comparisons of gene expression profiles. Island size: number of genes within each island.

| **NRN islands** | ***r* Medicago** | ***r* Lotus** | **Island size** | **log(nodules/root) Lotus** | **log(root/nodules) Medicago** |
| --- | --- | --- | --- | --- | --- |
| SRI_NRN0005 | 0.19 | 0.62 | 3 | -0.65 | 0.09 |
| SRI_NRN0060 | 0.14 | 0.61 | 4 | -0.96 | -0.74 |
| SRI_NRN0021 | -0.18 | 0.49 | 3 | -0.73 | 0.90 |
| SRI_NRN0034 | 0.04 | 0.46 | 3 | 0.16 | -0.44 |
| SRI_NRN0028 | 0.18 | 0.33 | 5 | 0.20 | 0.66 |
| SRI_NRN0068 | 0.06 | 0.31 | 5 | -0.24 | -0.12 |
| SRI_NRN0074 | -0.31 | 0.26 | 3 | -1.49 | -0.40 |
| SRI_NRN0044 | 0.19 | 0.24 | 3 | -0.46 | 0.26 |
| SRI_NRN0013 | -0.04 | 0.23 | 8 | 0.34 | -0.06 |
| SRI_NRN0075 | -0.03 | 0.23 | 3 | 0.53 | 0.10 |
| SRI_NRN0018 | 0.04 | 0.21 | 7 | 0.19 | -0.15 |
| SRI_NRN0014 | 0.02 | 0.20 | 3 | 0.74 | 4.06 |
| SRI_NRN0016 | 0.07 | 0.15 | 4 | -1.13 | -0.66 |
| SRI_NRN0035 | 0.19 | 0.14 | 4 | -0.22 | 0.14 |
| SRI_NRN0051 | 0.06 | 0.12 | 5 | 0.09 | -0.03 |
| SRI_NRN0038 | 0.00 | 0.10 | 7 | -0.16 | 0.30 |
| SRI_NRN0030 | 0.03 | 0.09 | 3 | 0.51 | 0.13 |
| SRI_NRN0056 | -0.23 | 0.08 | 4 | 0.22 | 0.08 |
| SRI_NRN0080 | -0.14 | 0.08 | 4 | -0.16 | -0.28 |
| SRI_NRN0079 | 0.07 | 0.07 | 4 | 0.25 | -0.08 |

**Supplemental table 4.** Top correlated NRN islands. The table is sorted by the Lotus correlation coefficient (*r*). *r*: average Pearson correlation coefficient for pairwise comparisons of gene expression profiles. Island size: number of genes within each island.

| **NRD islands** | ***r* Medicago** | ***r* Lotus** | **Island size** | **log(nodules/root) Lotus** | **log(root/nodules) Medicago** |
| --- | --- | --- | --- | --- | --- |
| SRI_NRD0027 | 0.43 | 0.88 | 5 | -4.36 | -1.63 |
| SRI_NRD0080 | 0.73 | 0.85 | 3 | -3.81 | -3.36 |
| SRI_NRD0087 | 0.71 | 0.74 | 3 | -4.08 | -3.30 |
| SRI_NRD0005 | -0.15 | 0.66 | 3 | -3.11 | -1.60 |
| SRI_NRD0017 | 0.71 | 0.64 | 4 | -2.75 | -2.91 |
| SRI_NRD0031 | -0.02 | 0.56 | 4 | -3.45 | -2.92 |
| SRI_NRD0047 | 0.21 | 0.49 | 3 | -2.70 | -0.49 |
| SRI_NRD0046 | 0.85 | 0.47 | 3 | -3.68 | -4.92 |
| SRI_NRD0045 | 0.82 | 0.37 | 3 | -4.01 | -3.62 |
| SRI_NRD0019 | 0.01 | 0.36 | 3 | -0.96 | -1.55 |
| SRI_NRD0044 | 0.22 | 0.36 | 5 | -1.47 | -2.56 |
| SRI_NRD0034 | -0.15 | 0.34 | 4 | -0.84 | -0.63 |
| SRI_NRD0043 | 0.77 | 0.34 | 3 | 2.30 | -1.35 |
| SRI_NRD0042 | 0.47 | 0.33 | 5 | -2.16 | -1.15 |
| SRI_NRD0072 | 0.49 | 0.28 | 4 | -0.86 | -3.09 |
| SRI_NRD0007 | 0.35 | 0.26 | 6 | -1.58 | -1.83 |
| SRI_NRD0006 | 0.67 | 0.20 | 3 | 1.55 | -2.10 |
| SRI_NRD0056 | -0.05 | 0.20 | 4 | -2.78 | -1.73 |
| SRI_NRD0024 | 0.55 | 0.17 | 4 | 2.99 | -2.93 |
| SRI_NRD0054 | 0.63 | 0.16 | 3 | -0.94 | -1.65 |

**Supplemental table 5.** Top correlated NRD islands. The table is sorted by the Lotus correlation coefficient (*r*). *r*: average Pearson correlation coefficient for pairwise comparisons of gene expression profiles. Island size: number of genes within each island.

| **gene ID** | **chromosome** | **start** | **end** | **strand** |
| --- | --- | --- | --- | --- |
| LotjaGi2g1v0394950 | chr2 | 89244457 | 89246244 | - |
| LotjaGi2g1v0440600 | chr2 | 93688296 | 93690318 | + |
| LotjaGi3g1v0414350 | chr3 | 81961144 | 81962772 | - |
| LotjaGi3g1v0449330 | chr3 | 86285726 | 86285971 | + |
| LotjaGi3g1v0449360 | chr3 | 86294832 | 86295050 | + |
| LotjaGi4g1v0376650 | chr4 | 75571048 | 75571728 | + |
| LotjaGi5g1v0359230 | chr5 | 67390287 | 67391829 | + |
| LotjaGi5g1v0359260 | chr5 | 67393483 | 67394919 | + |
| LotjaGi5g1v0359300 | chr5 | 67400904 | 67402147 | + |
| LotjaGi5g1v0359350 | chr5 | 67402703 | 67404660 | - |

**Supplemental table 6.** Manually curated genes.

# Supplemental references

Handa, Y., Nishide, H., Takeda, N., Suzuki, Y., Kawaguchi, M., and Saito, K. (2015). RNA-seq Transcriptional Profiling of an Arbuscular Mycorrhiza Provides Insights into Regulated and Coordinated Gene Expression in Lotus japonicus and Rhizophagus irregularis. Plant Cell Physiol. 56, 1490–1511.

Kelly, S., Mun, T., Stougaard, J., Ben, C., and Andersen, S.U. (2018). Distinct Lotus japonicus Transcriptomic Responses to a Spectrum of Bacteria Ranging From Symbiotic to Pathogenic. Front Plant Sci 9, 1218.

Munch, D., Gupta, V., Bachmann, A., Busch, W., Kelly, S., Mun, T., and Andersen, S.U. (2018). The Brassicaceae Family Displays Divergent, Shoot-Skewed NLR Resistance Gene Expression. Plant Physiol. 176, 1598–1609.
